# Environmental influences on visual hallucinations in Lewy body disease: the potential role of virtual reality

**DOI:** 10.3389/fnagi.2026.2005253

**Published:** 2026-10-28

**Authors:** Kazuhide Seo, Masayuki Hara, Selim Habiby Alaoui, Genko Oyama

**Affiliations:** 1 Department of Neurology, Saitama Medical University, Saitama, Japan; 2 Graduate School of Science and Engineering, Saitama University, Saitama, Japan

**Keywords:** dementia with Lewy bodies, environmental dependency, Lewy body disease, Parkinson’s disease, virtual reality, visual hallucinations

## Abstract

Lewy body disease (LBD), which includes Parkinson’s disease (PD) and dementia with Lewy bodies, is frequently accompanied by visual hallucinations, one of its most characteristic neuropsychiatric symptoms. These symptoms are clinically significant as they reduce quality of life, increase caregiver burden, and correlate with poorer prognosis. In clinical practice, visual hallucinations in LBD often appear under certain environmental conditions, including nighttime or low light settings, ambiguous shadows or patterns, and visual features such as furniture or decorations, suggesting environmental influence. However, this dependency has not been systematically characterized, and its mechanisms remain partially understood. Current evidence suggests that this phenomenon may arise from the interaction of reduced bottom-up visual input, excessive top-down influence, and impaired reality monitoring. Although pharmacological treatments remain crucial, adverse events and tolerability concerns often limit their use and increase discontinuation rates. Thus, investigating non-pharmacological approaches such as direct modifications to patients’ environment may provide an alternative approach to symptom management and treatment. In this Perspective article, we summarize the clinical features of visual hallucinations in LBD, review evidence regarding their environmental dependency, examine candidate pathophysiological mechanisms, and highlight virtual reality as a promising tool for mechanistic investigation and individualized environmental interventions.

## 1 Introduction

Lewy body disease (LBD), which encompasses Parkinson’s disease (PD) and dementia with Lewy bodies (DLB), is characterized by both parkinsonism and non-motor symptoms, including cognitive impairment and neuropsychiatric manifestations such as hallucinations and delusions (Onofrj et al., 2013; Gomperts, 2016; D'Antonio et al., 2022). Among these, visual hallucinations are a hallmark feature of LBD and are clinically significant, as they are associated with reduced quality of life, increased caregiver burden, and poorer prognosis (Onofrj et al., 2013; Gomperts, 2016; D'Antonio et al., 2022). These hallucinations occur in approximately 20–30% of patients with PD and 60–80% of those with DLB (Fénelon et al., 2000; de Maindreville et al., 2005; McKeith et al., 2017; Eversfield and Orton, 2019). In LBD, visual hallucinations encompass a broad spectrum of phenomena, ranging from minor experiences such as illusions, presence hallucinations, and passage hallucinations to complex visual hallucinations involving people, animals, insects, or objects (Onofrj et al., 2013; Gomperts, 2016; D'Antonio et al., 2022; Fénelon et al., 2011). In a study of 35 participants with LBD, D’Antonio et al. demonstrated substantial overlap among these phenomena; moreover, pareidolia has also been regarded as a manifestation of hallucination vulnerability within this disease spectrum (D'Antonio et al., 2022; Uchiyama et al., 2012). Collectively, these observations suggest that visual hallucinations in LBD should be understood not as an isolated symptom, but as manifestations of a broad spectrum of abnormal visual perception (Fénelon et al., 2011; Uchiyama et al., 2012).

Clinically, visual hallucinations in LBD appear to be influenced by the surrounding environment. Although nighttime or dimly lit settings are implicated, visual features such as floor and wall patterns and furniture have also been considered possible triggers (Gomperts, 2016; Fénelon et al., 2000; Taylor et al., 2019; Samudra et al., 2016; O'Brien et al., 2020; Yumoto and Suwa, 2021; Ishimaru et al., 2024). However, few studies have systematically examined this issue, and findings regarding the association between environmental conditions and visual hallucinations have been inconsistent. Among 21 patients with PD who experienced visual hallucinations, these hallucinations occurred at any time in 58%, only at night or in the dark in 29%, only in dim light in 10%, and only during the day or in bright light in 3% (Papapetropoulos et al., 2008). Another study reported that, among 13 patients with PD and visual hallucinations, 11 experienced hallucinations in the afternoon, evening, or night, and three identified dim lighting alone as the relevant condition (Diaz-Santos et al., 2021). Gu et al. further reported that 75.3% of patients perceived hallucinations as more likely to occur at night or under dim lighting (Gu et al., 2022). These variations across studies make it unclear whether visual hallucinations consistently depend on nighttime or low-light conditions.

Almost no quantitative studies have examined environmental factors other than illuminance, such as color temperature, wall and floor patterns, and furniture arrangement (Ishimaru et al., 2024). Current management strategies include lighting adjustment and the removal of furniture or decorative objects that may act as triggers (Taylor et al., 2019; O'Brien et al., 2020; Yumoto and Suwa, 2021); however, these are not based on direct evidence. The case report by Ishimaru et al., is the only one that specifically describes environmental modification based on assessment of the living environment. The researchers used photographs of the home environment to identify relevant factors in two patients with DLB, followed by improvement in both hallucinations and caregiver burden (Ishimaru et al., 2024). However, owing to the small sample size (two cases) and use of multimodal interventions, it could not be determined which environmental factors consistently induce or exacerbate hallucinations (Ishimaru et al., 2024).

The environmental dependency of visual hallucinations in LBD likely reflects the interaction of multiple mechanisms, including reduced bottom-up visual input, excessive top-down completion, and impaired reality monitoring—that is, the ability to distinguish internally generated percepts from externally derived stimuli. Consequently, internally generated percepts to be misidentified as real external stimuli (D'Antonio et al., 2022; Kurita et al., 2020; Bhome et al., 2023; Collerton et al., 2023). Bottom-up visual input refers to sensory information transmitted from the retina to visual cortical regions, and its vulnerability is supported by findings that hallucinations in LBD are associated with deficits in contour discrimination, contrast sensitivity, and color discrimination (Diaz-Santos et al., 2021; Weil et al., 2016), reduced occipito-temporal activation during recognition of ambiguous images in PD with hallucinations (Meppelink et al., 2009), and visual impairment or retinal nerve fiber layer thinning (Matsui et al., 2006; Lee et al., 2014; Visser et al., 2020). These findings suggest that dim environments may further degrade an already fragile visual system. However, this alone cannot explain why furniture, decorative objects, or wall and floor patterns may trigger hallucinations. It is presumed, that when visual input is ambiguous or degraded, top-down processes may compensate by filling in missing information on the basis of stored visual memories, thereby generating false percepts (Corradi-Dell'Acqua et al., 2015; Chiou and Lambon Ralph, 2016; Cosman et al., 2018). In patients prone to hallucination, this may result in excessive reliance on prior knowledge (Yao et al., 2014; Shine et al., 2015; Thomas et al., 2023), causing ordinary environmental features to become substrates for pareidolic misperception. Additionally, reality monitoring may be impaired in LBD (Ku et al., 2008; Buda et al., 2011), allowing such false percepts to be experienced as real hallucinations (Kurita et al., 2020; Barnes et al., 2003; Diederich et al., 2005). However, most available evidence comes from questionnaires or surrogate two-dimensional paradigms, such as pareidolia and blurred-image tasks (Uchiyama et al., 2012; Mehraram et al., 2022), which do not adequately capture hallucinations in real-life settings. Thus, experimental systems that can induce and quantify hallucinations while systematically manipulating environmental factors are needed.

Virtual reality (VR) may offer a promising approach for addressing these limitations. By providing controlled reproduction of real-world environments in laboratory or clinical settings, VR can elicit brain and behavioral responses that approximate those observed in everyday life (Riva et al., 2019). In PD, VR has been used in research on freezing of gait, another symptom strongly influenced by environmental context, because it allows environmental conditions to be systematically manipulated under controlled experimental settings (Bluett et al., 2019). Thus, for visual hallucinations, VR may similarly allow reconstruction of potentially provocative environments while allowing systematic manipulation of factors such as illuminance, color temperature, object arrangement, and background patterns. Because identical conditions can be presented repeatedly while quantitative measures such as gaze behavior, exploratory movements, reaction times, and movement trajectories are recorded, VR may provide a more objective and reproducible framework for investigating hallucination-related phenomena than subjective report alone. In this context, this Perspective reviews the available VR studies in hallucination research and discusses its potential utility for investigating the environmental dependency of visual hallucinations in LBD and informing future environmental interventions.

## 2 Literature search and study selection

A literature search was conducted in the PubMed database on March 10, 2026, to identify studies on hallucination research in LBD using VR technology. The following search query was used:

*((“Parkinson’s disease”[Title/Abstract] OR “Parkinson disease”[Title/Abstract] OR* “dementia with Lewy bodies”*[Title/Abstract] OR* “Lewy body disease”[Title/Abstract]) AND (hallucination*[Title/Abstract] OR psychosis [Title/Abstract]) AND (“virtual reality”[Title/Abstract] OR VR[Title/Abstract]))

This search yielded five articles. From these, we selected original research articles that used VR technology in studies of hallucinations in LBD. We screened the titles and abstracts and assessed the full texts of all five articles for eligibility. One case report (Tuena et al., 2022) was excluded because VR was used to evaluate spatial navigation and memory in a patient with hallucinations rather than to investigate hallucinations experimentally. Another study (Albani et al., 2009) was excluded because it described the same patient cohort as a subsequent longitudinal study (Albani et al., 2015), which provided extended follow-up and additional analyses. Ultimately, three studies (Albani et al., 2015; Onofrj et al., 2006; Albert et al., 2024) were included in this Perspective. The study selection process is illustrated in Figure 1.

**Figure 1:**
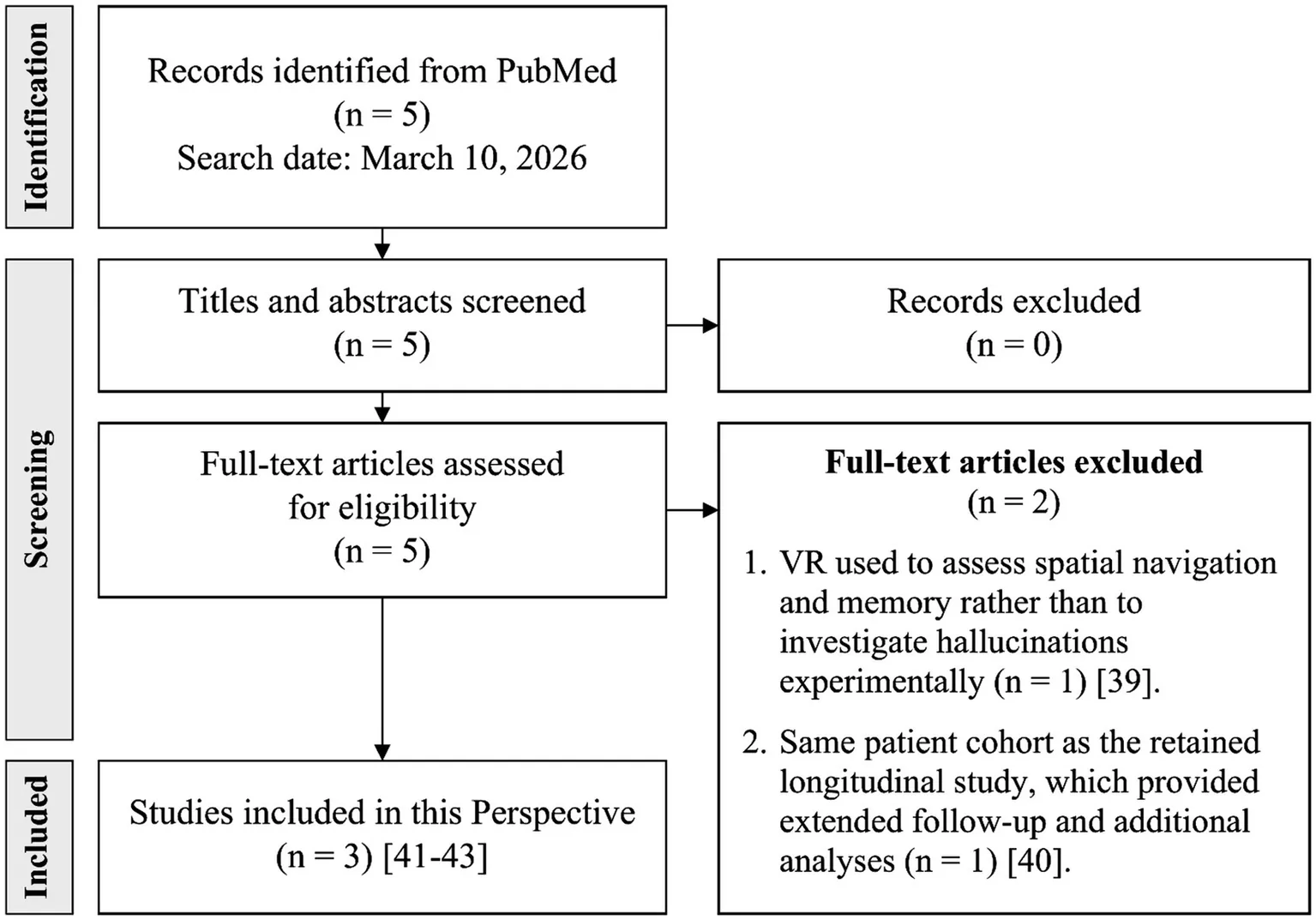
Flow diagram of the literature search and study selection. The PubMed search identified five articles, all of which underwent title and abstract screening and full-text assessment. Two articles were excluded for the reasons indicated, and three studies were included in this perspective. VR, virtual reality.

## 3 Utility of virtual reality for elucidating environmental dependency

The three selected studies suggest that VR may contribute to hallucination research in LBD in two main ways: by eliciting hallucination-like experiences within immersive virtual environments, and by enabling controlled experimental investigation of hallucination-related perceptual phenomena. Although the available evidence remains limited, these studies provide an initial basis for considering VR as a useful framework for investigating the environmental dependency of visual hallucinations in LBD. Onofrj et al., (2006), provided virtual environments resembling a supermarket, gymnasium, and kitchen to 23 patients with PD without a prior history of visual hallucinations using a head-mounted display (HMD), with each environment experienced for 35 min. They reported that six patients experienced the presence of persons, animals, or objects that were not presented in the virtual environment, and thus classified as hallucination events. The reported contents included complex visual hallucinations such as animals painted on the wall, a child sitting at a desk, bees flying around, and a gasoline pump placed in the kitchen. Visual hallucinations occurred during off periods of motor symptoms and lasted 3–25 s. The patients recognized that these images were incongruent with the context of the virtual environment and did not necessarily experience intense fear or astonishment, suggesting a phenomenological pattern resembling that of visual hallucinations observed in real-world LBD.

Albani et al., (2015), followed the same 23 patients with PD for 8 years after the VR experiment and reported their clinical outcomes. Remarkably, all six patients who had experienced visual hallucinations during VR subsequently developed visual hallucinations in real life, whereas none of the 17 patients who did not experience hallucinations during VR developed visual hallucinations during follow-up. Although the study did not describe whether the content of the hallucinations experienced in VR corresponded to that later experienced in real-world settings, these longitudinal findings suggest that incidental visual hallucinations during immersive VR may be associated with the subsequent emergence of clinically manifest visual hallucinations. If these findings can be replicated, they highlight the possibility that VR-based provocation paradigms could serve as a means of identifying patients with LBD who are at increased risk of developing clinically manifest hallucinations in the future. This possibility is clinically important because, in PD, hallucinations are associated with an increased risk of cognitive dysfunction (Aarsland et al., 2021; Hepp et al., 2013; Santos-Garcia et al., 2023), whereas in DLB, detecting such vulnerability before overt hallucinations emerge may contribute to earlier diagnosis, potentially even during the prodromal stage (McKeith et al., 2020).

Another, more controlled, study examining hallucination-related phenomena was conducted by Albert et al., (2024). In healthy participants, immersive HMD-based VR was combined with a robotic sensorimotor paradigm and the results showed that asynchronous tactile feedback increased presence hallucination ratings and selectively enhanced overestimation of the number of virtual human agents, whereas no comparable effect was observed for objects. Subsequently, this paradigm was translated into a home-based 2D online numerosity task without VR or robotic stimulation in patients with PD. The study found that patients with presence hallucinations overestimated the number of human agents more than patients without hallucinations, again without a corresponding effect for objects. These findings suggest that multisensory mismatch can experimentally amplify hallucination-related perceptual bias under controlled conditions. In PD, although this task may have elicited hallucination-related perceptual phenomena, the greater overestimation could reflect cognitive dysfunction, including biased or inaccurate numerosity estimation, rather than hallucination-related perceptual disturbance alone, and should therefore be interpreted cautiously (Aarsland et al., 2021; Hepp et al., 2013; Santos-Garcia et al., 2023).

## 4 Discussion

Visual hallucinations in LBD are often described clinically as context-sensitive phenomena, but the evidence reviewed here suggests that this environmental dependency is not merely an anecdotal bedside impression. Rather, it reflects a meaningful interaction between disease-related vulnerability in visual and cognitive systems and external environmental features (D'Antonio et al., 2022; Ishimaru et al., 2024; Kurita et al., 2020; Bhome et al., 2023; Collerton et al., 2023). In this framework, dim illumination, ambiguous visual scenes, and visually complex objects may actively shape hallucination risk by further degrading already impaired sensory input and increasing reliance on internally generated predictions (D'Antonio et al., 2022; Ishimaru et al., 2024; Diaz-Santos et al., 2021; Kurita et al., 2020; Bhome et al., 2023; Collerton et al., 2023; Weil et al., 2016; Meppelink et al., 2009; Matsui et al., 2006; Lee et al., 2014; Visser et al., 2020; Corradi-Dell'Acqua et al., 2015; Chiou and Lambon Ralph, 2016; Cosman et al., 2018; Yao et al., 2014; Shine et al., 2015; Thomas et al., 2023; Ku et al., 2008; Buda et al., 2011; Barnes et al., 2003; Diederich et al., 2005; Mehraram et al., 2022; Zarkali et al., 2019). This perspective illustrates the connection between everyday clinical observations and contemporary models of hallucination pathophysiology.

The potential environmental dependency of visual hallucinations also highlights the possibility of non-pharmacological interventions in targeting modifiable environmental factors. Current management emphasizes exclusion of acute reversible causes such as delirium, infection, dehydration, and metabolic disturbance, along with assessment of pain, constipation, fecal impaction, sleep disturbance, and potentially contributing medications (McKeith et al., 2017; Goldman and Holden, 2014; Pirker et al., 2026). Pharmacological treatment remains important, and cholinesterase inhibitors such as donepezil may reduce psychiatric symptoms including visual hallucinations in DLB (Mori et al., 2012; Mori et al., 2015; Mori et al., 2024). However, these treatments are not universally tolerated, and antipsychotic use in LBD remains particularly challenging owing to the risk of antipsychotic sensitivity, worsening parkinsonism, sedation, cognitive decline, and increased mortality (McKeith et al., 2017; Armstrong and Weintraub, 2017; Ballard et al., 1998; McKeith et al., 1992). In this context, environmental modification deserves greater attention not only as a supportive measure but also as a rational and potentially mechanism-informed component of care (Taylor et al., 2019; O'Brien et al., 2020; Yumoto and Suwa, 2021; Ishimaru et al., 2024; Connors et al., 2018).

However, the current evidence base remains inadequate. Although hallucinations are widely believed to occur more readily at night or under dim lighting, the available studies remain limited and inconsistent (Papapetropoulos et al., 2008; Diaz-Santos et al., 2021; Gu et al., 2022). The contribution of more specific environmental features, such as color temperature, spatial layout, wall or floor patterns, and the visual density of domestic objects is poorly understood (Ishimaru et al., 2024). Adjusting lighting and removing triggering visual stimuli are common clinical practices; however, they are supported by minimal direct evidence and remain poorly defined in operational terms (Taylor et al., 2019; O'Brien et al., 2020; Yumoto and Suwa, 2021; Connors et al., 2018). The case report by Ishimaru et al. (Ishimaru et al., 2024) is therefore crucial as it demonstrates clinical applications and highlights the scarce systematic evidence currently available.

A major reason for this gap is methodological. Most studies have relied on questionnaires, retrospective descriptions, or laboratory paradigms using two-dimensional ambiguous images (Uchiyama et al., 2012; Papapetropoulos et al., 2008; Diaz-Santos et al., 2021; Gu et al., 2022; Mehraram et al., 2022). These approaches have advanced the field, but they do not adequately capture the real-world interaction between perceptual uncertainty, environmental context, and hallucination emergence. In this regard, VR appears particularly promising (Riva et al., 2019; Bluett et al., 2019). Immersive virtual environments can induce hallucination-like experiences in PD and may also help identify patients with LBD who are at risk of developing clinically manifest hallucinations in the future (Albani et al., 2015; Onofrj et al., 2006). In addition, multisensory mismatch within VR-based paradigms can modulate hallucination-related phenomena such as presence hallucinations and over-attribution of human agents (Albert et al., 2024). Although these studies did not directly test the environmental dependency of visual hallucinations in LBD, they provide evidence that controllable immersive virtual environments can interact with hallucination-prone perceptual neural systems in many measurable ways (Onofrj et al., 2013; Albani et al., 2015; Albert et al., 2024).

However, the available VR studies have examined incidental hallucination-like experiences (Albani et al., 2015; Onofrj et al., 2006), presence hallucinations, or related perceptual biases (Albert et al., 2024), without directly testing how specific environmental features contribute to complex visual hallucinations. Future studies should therefore use immersive VR to systematically manipulate illuminance, color temperature, background ambiguity, wall and floor patterns, furniture arrangement, and visual crowding. These studies should examine whether the effects of environmental factors differ between PD and DLB and how these effects vary with disease severity, visual dysfunction, cognition, and medication state. Longitudinal follow-up is also needed to determine whether VR-based measures can predict the subsequent emergence of clinically manifest hallucinations.

## 5 Conclusion

Visual hallucinations in LBD may reflect interactions between disease-related perceptual and cognitive vulnerabilities and the surrounding environment. VR offers a promising experimental approach to examining these interactions under controlled and reproducible conditions. With further clinical validation, this approach could inform individualized environmental interventions for hallucination management.

## Data Availability

The original contributions presented in the study are included in the article/supplementary material, further inquiries can be directed to the corresponding author.
